# The consequence of fetal ethanol exposure and adolescent odor re-exposure on the response to ethanol odor in adolescent and adult rats

**DOI:** 10.1186/1744-9081-5-3

**Published:** 2009-01-15

**Authors:** Amber M Eade, Paul R Sheehe, Juan C Molina, Norman E Spear, Lisa M Youngentob, Steven L Youngentob

**Affiliations:** 1 Department of Neuroscience and Physiology, State University of New York Upstate Medical University, Syracuse, NY, USA; 2 Department of Psychology, Binghamton University, Binghamton, NY, USA; 3 State University of New York Developmental Exposure Alcohol Research Center, Syracuse & Binghamton, NY, USA

## Abstract

**Background:**

An epidemiologic predictive relationship exists between fetal ethanol exposure and the likelihood for adolescent use. Further, an inverse relationship exists between the age of first experience and the probability of adult abuse. Whether and how the combined effects of prenatal and adolescent ethanol experiences contribute to this progressive pattern remains unknown. Fetal ethanol exposure directly changes the odor attributes of ethanol important for both ethanol odor preference behavior and ethanol flavor perception. These effects persist only to adolescence. Here we tested whether adolescent ethanol odor re-exposure: (Experiment 1) augments the fetal effect on the adolescent behavioral response to ethanol odor; and/or (Experiment 2) perpetuates previously observed adolescent behavioral and neurophysiological responses into adulthood.

**Methods:**

Pregnant rats received either an ethanol or control liquid diet. Progeny (observers) experienced ethanol odor in adolescence via social interaction with a peer (demonstrators) that received an intragastric infusion of either 1.5 g/kg ethanol or water. Social interactions were scored for the frequency that observers followed their demonstrator. Whole-body plethysmography evaluated the unconditioned behavioral response of observers to ethanol odor in adolescence (P37) or adulthood (P90). The olfactory epithelium of adults was also examined for its neural response to five odorants, including ethanol.

**Results:**

Experiment 1: Relative to fetal or adolescent exposure alone, adolescent re-exposure enhanced the behavioral response to ethanol odor in P37 animals. Compared to animals with no ethanol experience, rats receiving a single experience (fetal or adolescent) show an enhanced, yet equivalent, ethanol odor response. Fetal ethanol experience also increased olfactory-guided following of an intoxicated peer. Experiment 2: Combined exposure yielded persistence of the behavioral effects only in adult females. We found no evidence for persistence of neurophysiological effects in either sex.

**Conclusion:**

Fetal ethanol exposure influences adolescent re-exposure, in part, by promoting interactions with intoxicated peers. Re-exposure subsequently enhances ethanol odor responsivity during a key developmental transition point for emergent abuse patterns. While persistence of behavioral effects occurred in females, the level of re-exposure necessary to uniformly yield persistence in both sexes remains unknown. Nonetheless, these results highlight an important relationship between fetal and adolescent experiences that appears essential to the progressive pattern of developing ethanol abuse.

## Background

Clinical and epidemiological studies have demonstrated a strong predictive relationship between fetal ethanol exposure and the risk for abuse in adolescence and early adulthood. Fetal exposure is, perhaps, the best predictor of ethanol abuse in this "at risk" age group, surpassing even family history of alcohol related problems [1-5]. There is also an inverse correlation between the age of first experience and the likelihood of continued abuse [4,5]. Little is known, however, of the underlying biological factors contributing to the progressive pattern.

Much is known regarding what the human and animal fetus can learn behaviorally about chemosensory stimuli, including ethanol, as a consequence of fetal exposure. Indeed, such learning may be a fundamental feature of all mammalian species because it is important (from a survival standpoint) for the pre-weanling animal to accept and be attracted to the food sources consumed by the mother [6]. In humans there is evidence from infant testing that the fetus has the ability to detect and learn odor information via the mother's diet [e.g., [7-9]]. Thus, the gaining of odor and flavor information by the human fetus is likely to affect the responsiveness to the sensory qualities of a fetal exposure stimulus [10], such as ethanol. Indeed, studies focusing on the effects of prenatal ethanol exposure on later responsiveness to and acceptance of the drug have demonstrated that the fetus can acquire a memory for ethanol's chemosensory attributes [11-18].

One contributing mechanism to the above, fetal ethanol experience has been shown to tune the olfactory systems response specifically to ethanol odor. Rats exposed to ethanol throughout gestation display an enhanced neural and behavioral response to ethanol odor [19]. More importantly, Youngentob and Glendinning [submitted] have recently shown that the alterations in the behavioral response to ethanol odor modify the odor attributes of ethanol that are key to both a preference for its odor and flavor perception. That is, the effect of prenatal exposure on the response to ethanol odor not only significantly predicts the observed prenatal effect on enhanced ethanol intake but, more importantly, a significant proportion of the enhanced ethanol intake effect can be *directly *attributed to the enhanced behavioral response to ethanol odor.

Interestingly, these consequences although absent in adults [19,20] persist into adolescence. That is, fetal exposure both enhances the adolescent functional response to ethanol odor consistent with that observed in infant animals [21] and enhances ethanol intake [22-24]. Taken together, these observations suggest that adolescence is a key developmental transition point for perpetuating the effects of fetal ethanol exposure on odor-induced plasticity and its contribution to later acceptance patterns of the drug.

The present study, therefore, focused on an examination of the consequence of fetal ethanol exposure and brief adolescent odor re-exposure with respect to two questions. In Experiment 1, we tested the hypothesis that relative to fetal ethanol exposure alone, adolescent re-exposure will augment the behavioral response to ethanol odor in animals tested in adolescence. Experiment 2 tested the hypothesis that combined fetal and adolescent exposure will yield persistence of the known infant and adolescent behavioral and neurophysiological into adulthood.

## Methods

### General note

A total of 288 experimental rats were utilized in these studies. Animals were housed at SUNY Upstate Medical University in a temperature and humidity controlled environment on a fixed 12 hour light-dark cycle. All treatments and testing were completed in accordance with the guidelines set by the SUNY Upstate Medical University's Institutional Animal Care and Use Committee.

### Treatment of pregnant dams

For both experiments, Long-Evans female rats (Harlan-Sprague Dawley, Indianapolis, IN) were weighed and placed into weight-matched groups of three dams each (*a block for analytic purposes*) on gestational (G) day 5. A total of six triads were used for each experiment. Within each triad, dams were randomly assigned to one of the three maternal treatments: an experimental dam (ET) fed ethanol in a liquid diet, a pair-fed (PF) dam or a free-choice dam (FC).

For the ET treatment group, ethanol was administered through an *ad-libitum *liquid diet (L10251, Research Diets, NJ) supplemented with increasing levels of ethanol (2.2% v/v G6–G8, 4.5% v/v G9–G10, 6.7% v/v) [e.g., [19,20]]. The 6.7% diet provided the dams with 35% of their daily calories coming from ethanol, emulating a moderate ethanol intake [25,26]. Peak blood alcohol levels reach approximately 150 mg/dl on the evening of G17 [19,27]. Increasing the ethanol levels over time served to wean animals onto the diet such that we achieved the highest level of dietary exposure just before the time that olfactory neurons in rat fetuses (G14) begin to transduce sensory information [28] and before these neurons make synaptic connections with the olfactory bulb (G14–15) [29].

Both the PF and FC control groups were fed an iso-nutritive liquid diet with maltose/dextrin substituted for the calories derived by ethanol (L10252, Research Diets, NJ). The PF dams were restricted to the caloric intake of their respective weight-matched ethanol dam within each block to control for potential nutritional deficits that might arise due to the ET dam voluntarily consuming less diet. The PF dams had access to the same volume of liquid diet that the respective ET dams consumed on the previous day. FC dams had *ad-libitum *access to liquid diet and water throughout gestation.

Within 24 hours of birth, litters from the three maternal treatment groups were fostered to surrogate dams that received *ad-libitum *access to food and water throughout gestation. Litters were sexed and culled to 10 pups on the morning of postnatal day (P) 2 with the restriction that litters contained no fewer than 4 pups of either sex.

### Re-exposure of adolescent animals

Adolescent re-exposure was accomplished using a social transmission of food odor preference paradigm. This method was chosen based on its requisite ability to transmit dietary information via the olfactory system. It is important to note that smelling a food on the breath of a conspecific leads to much stronger alterations in later responsiveness to a food source than mere familiarity with the substance [30-32].

**Figure 1:**
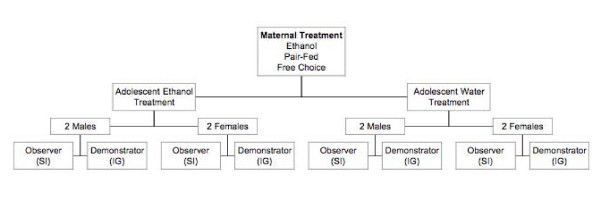
**Allocation of animals for the adolescent social transmission paradigm**. SI = Exposure via social interaction. IG = Exposure via intragastric infusion. See text for details.

At weaning (P21), eight pups (4 male; 4 female) from each maternal treatment were separated and randomly allocated into cages of 2 same sex siblings that remained together for the entirety of the experiment (Fig. 1). One pair from each ET, PF and FC litter was allocated to either the water experience or ethanol experience groups. Within each cage of siblings, one animal was further randomly selected as the demonstrator and the other the observer. As such, an animal within a pair could be a demonstrator for either ethanol or water and likewise the observer could experience the same. Thus, with this exposure design we were able to produce the appropriate groups of animals necessary to test specific hypotheses related to pre- and postnatal exposure.

Adolescent experience, with either ethanol or water, began on P29 and occurred 4 times, 48 hours apart (on P29, P31, P33, & P35). On each exposure day the pairs were separated for 1 hr prior to social interaction (SI) (i.e., the observer remained in the home cage and demonstrator was removed). Thirty minutes into the separation, the demonstrator was intragastrically (i.g.) infused with either a subnarcoleptic dose of 1.5 g/kg ethanol (ethanol demonstrator) (a dose that has been found to increase odor preference and consumption of ethanol by naïve adolescent observers) or the equivalent volume of tap water (water demonstrator) [e.g., [33,34]]. Infusion was accomplished using a polyethylene cannula (PE-10) attached to a 5 cc syringe. While holding the animal by the scruff of the neck, the free end of the cannula was inserted through the oral cavity and into the stomach. The entire process took approximately 20 seconds per animal and has been shown to yield minimal evidence of stress [13,35]. Thirty minutes after the infusion, the demonstrator was returned to its home cage for 30 minutes of social interaction with the observer. Following the interaction, animals were separated for 4 hours. This period of separation ensured ethanol had cleared the ethanol demonstrator's system prior to being returned to the home cage with the observer sibling for overnight housing.

### Recording of social interaction behavior

The first 30-min social interaction period was digitally taped using an iSight webcam (Apple Computers Inc., Cupertino, CA) and Security Spy software (Ben Software, ). An iMac computer (Apple Computers Inc., Cupertino, CA) operated the Security Spy Program and recorded images simultaneously from each of two iSight webcams at 30 frames/sec. Each camera recorded two cages of social interactions concurrently. Therefore, all four social interactions from one maternal treatment group (Fig. 1) were completed in the single 30-minute period. Upon completion of recording, the videos were transferred onto an external hard-drive for storage and later analysis.

For each pair of adolescent animals, only the first 10 minutes of the 30-min social interaction was viewed for scoring purposes (animals had a tendency to fall asleep after 10 min.). Regarding social interaction behaviors, previous studies have measured the occurrence of several forms of activity such as rearing, self-grooming, following, sniffing, crawling under/over, social grooming, nape attacks, pinning, and cuddling [35,36]. However, the specific focus of the present study was on the olfactory response to ethanol odor. Therefore, we directed our evaluation to the chemosensory-guided behavior of "following". In this respect, we suggest that the easily observable behavior of "following" is driven by exploratory sniffing, which, in and of itself, cannot be quantified with video. In other words, following behavior occurs, at least in part, due to interest in an odor, which is initiated through active exploratory sniffing. More importantly, examination of our following behavior demonstrated a proportional relationship with the amount of time spent in mouth-to-mouth contact, a measure that has been previously shown to facilitate social transmission of food information [31]. Thus, the number of times an observer followed their respective demonstrator peer was manually recorded for the first 10 minutes of each pairs' first social interaction session.

### Assessment of reflexive sniffing behavior

The focus of our behavioral analysis was directed toward examining the unconditioned reflexive sniffing response to ethanol odor as a consequence of fetal and/or adolescent ethanol exposure. Alterations in stimulus-induced sniffing in response to ethanol odor were monitored using whole-body plethysmography [19,37]. Briefly, the testing chamber (and matching reference chamber) consisted of a 1.3 L Plexiglas cylinder that permitted rapid odorant delivery and clean out. A computer controlled stimulus generation and presentation, as well as data collection. Stimuli were generated using standard flow-dilution olfactometry.

Each adolescent (P37) or adult (P90) observer animal was tested once. A testing session began with 40 air-only trials as a habituation period. Following habituation, air and ethanol odor trials were presented using a fixed 6s inter-trial interval schedule. Stimuli were presented randomly in blocks of 10 air and 10 odor stimuli. Five different concentrations of ethanol odor (0.313%, 0.625%, 1.25%, 2.5% and 5% of vapor saturation at 20°C) were presented as odor stimuli. Each stimulus concentration was presented for one complete block of trials and odorant concentration between blocks was incremented in an ascending order.

### Behavioral response index

Previously it has been demonstrated that: (1) the profile of air movement (i.e., sniffing) is a complex response pattern that varies with odorant stimuli [38]: and (2) more importantly, although sniffing patterns can be deconstructed into a large number of descriptive variables (e.g., sniff volume, flow-rate and number), knowledge about any single variable is insufficient to evaluate the meaning of the behavioral response to odorant stimuli. By contrast, however, this complex pattern of behavior can be described and evaluated using a univariate measure that incorporates the derived variables along with their corresponding weightings [19,37,38]. To construct a "Composite Reflexive Sniffing Index" the odorant-induced sniffing response pattern for each stimulus presentation was first deconstructed into 14 response measures: sniff frequency; the number of inspiratory and expiratory sniffs; the duration, volume, average flow rate, and peak flow rate of an inspiratory and expiratory sniff; the total inspiratory and expiratory volume; and the total apneic duration [19,37]. Next, principle components analysis (PCA) was used to reduce the 14 derived characteristics (i.e., dimensions) of each hypothesis specific data set to a fewer number of uncorrelated dimensions [19,37]. Recall that, each animal contributes a 14 × 5 data matrix to the overall data set; specifically, 14 variables at each of the 5 concentrations of ethanol. The PCA in conjunction with least square multiple regression [39] was used, in turn, to reduce each animal's 14 × 5 data matrix to a single response variable at each of five concentrations of ethanol (i.e., a 1 × 5 matrix) [19]. Finally, to construct the relevant "Composite Reflexive Sniffing Index" for each animal that incorporated the behavioral response across all concentrations tested, we estimated the coefficient for each of the five stimulus response measures (i.e. five behavioral response measures from the PCA) in a regression model. The composite index value for an individual animal was the linear summation of the constant from the regression analysis plus the animal's respective PCA value at each concentration of odorant tested times its' respective estimated coefficient.

### Optical recording of odorant-induced epithelial activity

Briefly, using optical recording methods and a voltage-sensitive dye (di-4-ANEPPS), we assessed the response of both the septum and turbinate olfactory epithelium (OE) to odorant stimulation [for technical details see [40]]. Each tissue was imaged onto a 120 × 120 pixel array of a Dalsa 12-bit digital CCD Camera (Dalsa, Waterloo, Ontario, Canada) [e.g., [19,41]]. To expose the OE for recording, the right nasal cavity of a decapitated rat was split to expose the septum and turbinates. Each piece of tissue was soaked in di-4-ANEPPS, rinsed with saline, and then placed in a clear top Delrin chamber. The chamber contained both stimulus input and output ports. The input port allowed odorant or air to be passed through the chamber and pulled across the OE from external naris to nasopharynx by a vacuum at 200 cc/min. A single concentration of 6 different odorants was presented individually at levels known to produce sizeable response patterns in the rat [e.g., [19,40,41]]. The concentrations of the odorants were as follows: 0.8% amyl acetate, 0.8% propyl acetate, 4% heptanal, 33% ethanol, 30% carvone, and 45% ethylacetoacetate of vapor saturation at 23°C. Amyl acetate was presented at the beginning and end of each session, serving as a standard stimulus for correction of tissue responsiveness over time. The raw responses were corrected for baseline shifts due to photo bleaching as well as adjusted for the levels of background fluorescence.

For each stimulus presentation, we recorded the response of the OE in terms of 2 measures of response magnitude (average and peak response heights) and 3 temporal measures (latency: start, peak and end times) [19]. For each tissue evaluated, the overall neural response of the OE was characterized by encapsulating the five response measures (i.e., response dimensions) into a smaller number of uncorrelated dimensions using a PCA. *A priori *[19], the first factor of the PCA was used to represent a single measure of the animals' neural response to each specific test odorant.

### Experimental procedure

In Experiment 1, we restricted our evaluation of adolescent odor re-exposure to only the adolescent behavioral response to ethanol odor. In this respect, we did not evaluate for an augmentation of a neurophysiologic effect since it is known that enhancement of odorant-induced mucosal activity requires more extensive stimulation over time than that used in the present study [42-44]. Nonetheless, this did not obviate the possibility that the postnatal experience obtained through the social interaction procedure may serve to stabilize (i.e., perpetuate) the prenatal neurophysiologic effect [19] into adulthood. Therefore, in Experiment 2 each adult animal was behaviorally tested and at the completion of testing, these same animals were killed and their mucosal response to odorant stimulation assessed.

### Analytic strategy

In this study, we were not interested in testing the reliability of observed overall differences related to exposure design main effects. Rather, based on previous work [19], for both experiments we tested a set of *a priori *hypotheses (using appropriately adjusted error terms). In this respect, for each specific hypothesis there was a specifically relevant effect to be evaluated such that what was most relevant to test one hypothesis was not most relevant to test another.

## Results

### Experiment 1: Adolescent behavioral assessment

#### Reflexive sniffing response to ethanol odor

**Figure 2:**
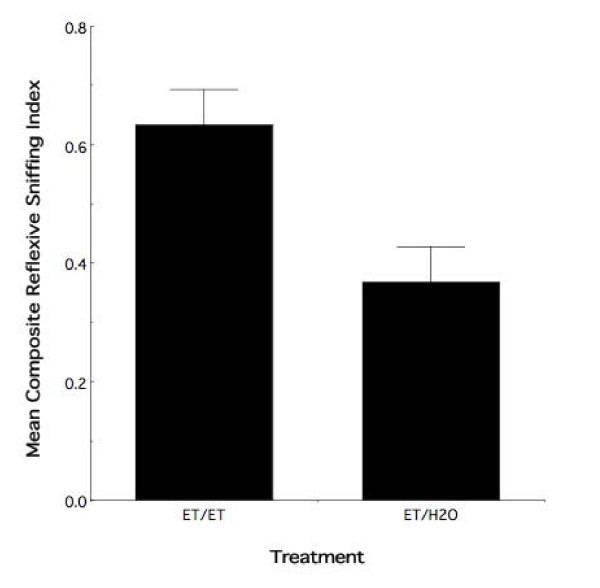
**Adolescent ethanol re-exposure augments the behavioral effect of prior fetal exposure**. Mean composite reflexive sniffing index values (± sem) for all adolescent observer animals that received prenatal ethanol exposure as a function of adolescent treatment. Relative to prenatal experience alone, animals with subsequent ethanol re-exposure in adolescence display altered responses to ethanol odor at this age. ET/ET = prenatal and adolescent ethanol exposure. ET/H2O = prenatal ethanol exposure and adolescent water exposure.

The goal of the experiment was to test the hypothesis that re-exposure to ethanol odor during adolescence further enhances the known adolescent behavioral response resulting from fetal experience with the drug [21]. To test this precise question, we evaluated the consequence of adolescent ethanol odor re-exposure, relative to fetal ethanol exposure alone, on the behavioral response to ethanol odor. In each of the analyses below the creation of the Composite Reflexive Sniffing Indexes were each based on the finding that the second factor of the PCA analyses met our criterion for variable selection from the multiple regression analyses (*F *≥ 2.0: [39]). Figure 2 illustrates the mean Composite Reflexive Sniffing Index (± sem) of all observer animals receiving prenatal ethanol experience (all ET animals) as a function of subsequent adolescent odor experience (i.e., interaction with an ethanol or water demonstrator). The results demonstrate that the response to ethanol odor significantly differed between animals with adolescent re-exposure and those with prenatal experience alone (*F*_1,16 _= 8.40, *p *< 0.01). There was no evidence of a differential sex effect (*F*_1,16 _= 0.01, *p *> 0.85).

**Figure 3:**
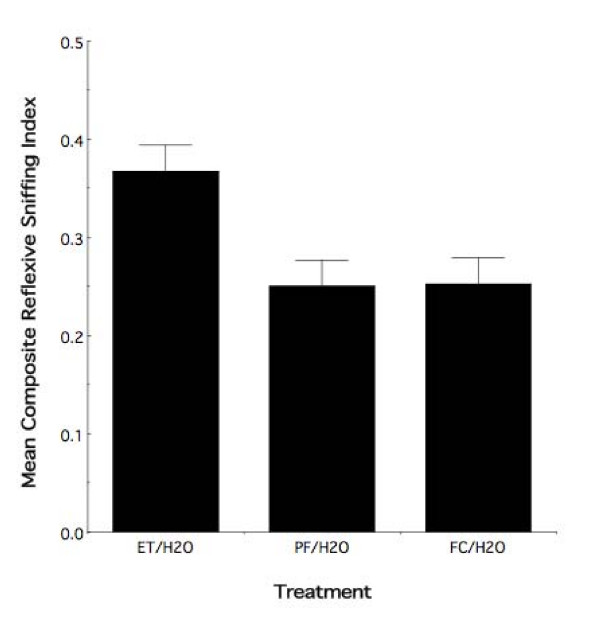
**Fetal ethanol exposure alters the behavioral response to ethanol odor in adolescence**. Mean composite reflexive sniffing index values (± sem) for all adolescent animals exposed to water infused demonstrator in adolescence as a function of maternal treatment. Ethanol animals with no further exposure to the drug display an altered behavioral response to ethanol odor when compared to pair-fed and free choice controls. ET/H2O = prenatal ethanol exposed animals with water exposure in adolescence. PF/H2O = pair-fed animals with water exposure in adolescence. FC/H2O = free choice animals with water exposure in adolescence.

The test of the foregoing hypothesis was predicated on the previous finding that gestational exposure to ethanol results in an altered olfactory response that persists into adolescence [21]. The prior result not withstanding, two issues require consideration in order to fully interpret the meaning of the above result: (1) although unlikely, prenatal ethanol exposure may not have resulted in the presence of an altered olfactory response at our age of testing in the present study; and (2) although, on average, adolescent re-exposure appeared to alter the response of fetal experience alone, the combined effect of fetal and adolescent experience may be no different than the effect of adolescent experience alone. Regarding the first consideration, Figure 3 illustrates the mean Composite Reflexive Sniffing Index (± sem) for animals having received interaction with a water demonstrator in adolescence as a function of maternal treatment. As can be seen in this figure, relative to PF and FC controls, prior fetal experience alters the adolescent behavioral response to ethanol odor. Randomized-blocks ANOVA demonstrated an overall effect of maternal treatment (*F*_2,27 _= 6.06, *p *< 0.007) with no evidence of a sex effect (*F*_1,27 _= 0.95, *p *> 0.30). Thus, as expected based on previous work, fetal experience with ethanol results in an altered response to ethanol odor in adolescence. Moreover, with regard to Figure 2, adolescent re-exposure enhances this response.

**Figure 4:**
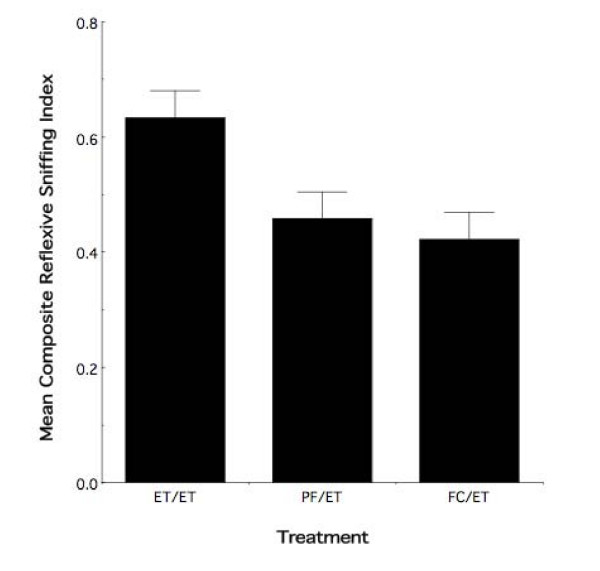
**Combined ethanol exposures results in an altered behavioral response as compared to adolescent exposure alone**. Mean composite reflexive sniffing index values (± sem) for all adolescent animals exposed to ethanol odor in adolescence as a function of maternal treatment. Relative to pair-fed and free choice control animals that received ethanol odor exposure during adolescence, adolescent re-exposure to ethanol alters the behavioral response in animals with prior fetal ethanol experience. ET/ET = animals with both fetal and adolescent ethanol exposure. PF/ET = pair-fed animals with ethanol exposure in adolescence. FC/ET = free choice animals with ethanol exposure in adolescence.

As noted in the second interpretive consideration, above, the behavioral result in Figure 2 might represent the consequence of the most proximate exposure. Figure 4 illustrates the mean (± sem) index values for all observer animals exposed to ethanol odor during adolescence as a function of prior maternal treatment. The behavioral results illustrate a clear overall difference in the response to ethanol odor in the adolescent ethanol exposed ET group as compared to the adolescent ethanol exposed PF and FC controls. Randomized-blocks ANOVA indicates an overall significant effect of prenatal treatment (*F*_2,27 _= 5.65; *p *< 0.009) on the response to ethanol odor in adolescent animals that had received ethanol odor experience during adolescence. There was no evidence of a differential sex effect (*F*_1,27 _= 1.41; *p *> 0.20).

**Figure 5:**
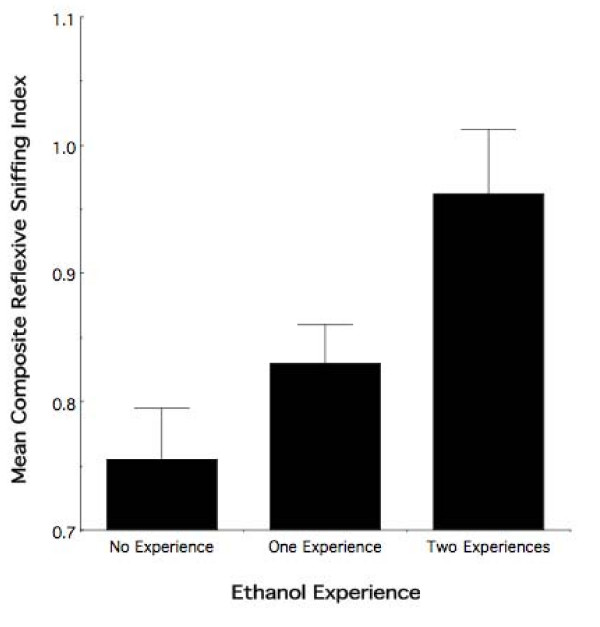
**The behavioral consequence of ethanol odor exposure appears to be cumulative**. Mean composite reflexive sniffing index values (± sem) of adolescent observers as a function of the number time periods in development during which ethanol experience occurred (i.e., fetal and/or adolescent). Note the graded effect with increasing experience. No ethanol experience = free choice and pair-fed animals with water experience in adolescence. One ethanol experience = all animals having received one developmental time point of experience with ethanol (i.e., free choice and pair-fed observer animals with ethanol odor experience in adolescence and prenatal ethanol observer animals with water experience in adolescence. Two ethanol experiences = observer animals receiving both prenatal and adolescent ethanol experience.

The foregoing results demonstrate that, indeed, the combined consequences of fetal and adolescent ethanol odor experience exceed the behavioral effects observed with either fetal or adolescent experience alone. Figure 5 illustrates a subsidiary exploratory analysis that further emphasizes the extent to which the varying degrees of ethanol experience influences the behavioral response to ethanol odor in adolescence. To construct this figure, we combined all observer animals having received no exposure to ethanol (i.e., FC and PF animals with water experience in adolescence) into a single "no ethanol experience" group. A preliminary exploratory analysis of these groups revealed no differential effect of treatment and no evidence of a sex effect (see Fig 3). Similarly, analysis of all animals having received one time period of experience with ethanol (i.e., FC and PF observer animals with ethanol odor experience in adolescence [Fig. 4] and ET observer animals with water experience in adolescence [Fig. 2]) revealed no evidence of a differential behavioral response. The third and final group consisted of the observer animals receiving both pre-natal and adolescent ethanol experience, forming the "two ethanol experiences" group. Note that, unlike the previous figures, the groups are not composed of equal animal numbers. Further, the scale of the figure has changed based on the new and unbalanced groups used in the creation of the Composite Sniffing Index for this analysis exploratory analysis.

As can be seen in Figure 5, there is a graded differential behavioral response to ethanol odor as a function of the number of developmental time points during which ethanol experience occurred. Those animals receiving both fetal and adolescent experience with ethanol show a differential behavioral response to ethanol odor relative to those animals with either one time period of experience (i.e., fetal or adolescent) or no experience with ethanol. Moreover, the behavioral response to ethanol odor is different in animals receiving one time period of experience relative to animals with no ethanol experience. Exploratory randomized-block ANOVA highlights that the number of developmental time periods during which ethanol experience occurred effects the adolescent behavioral response to ethanol odor (*F*_2,63 _= 3.61; nominal *p *< 0.03). There was no evidence of a sex effect (*F*_1,63 _= 0.83; nominal *p *> 0.35).

#### Social interaction behavior

**Figure 6:**
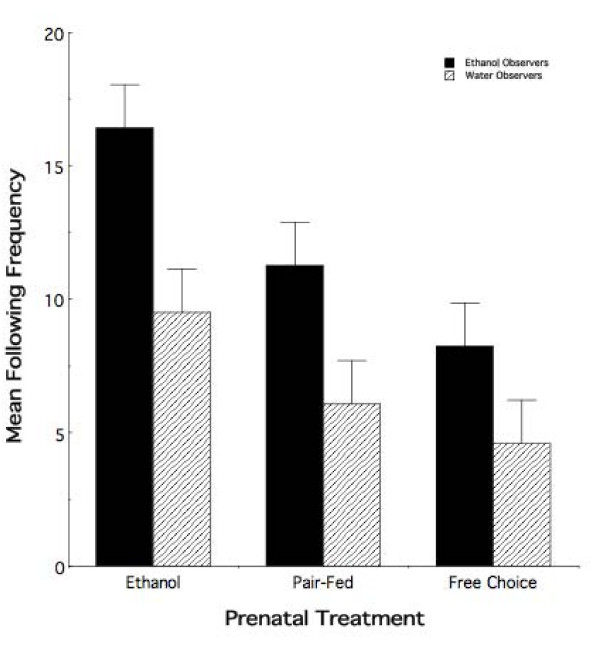
**Prenatal ethanol exposure leads to increases in the frequency of following an intoxicated peer**. Mean following frequency (± sem) for adolescent animals observing either an intoxicated or non-intoxicated peer in adolescence as a function of prenatal treatment. Ethanol animals display a specific increase in following behavior in response to an intoxicated peer relative to pair-fed and free choice controls.

Figure 6 illustrates the mean (± sem) number of times observers from each of the three prenatal treatment groups followed an intoxicated or non-intoxicated peer during the first 10 minutes of the first 30-minute social interaction. Randomized-blocks ANOVA demonstrated significant overall effects of both prenatal (*F*_2,60 _= 8.25; *p *< 0.0007) and adolescent (*F*_2,60 _= 15.44; *p *< 0.0003) treatments with no evidence of a differential sex effect (*F*_1,60 _= 0.22; *p *> 0.60). While we did not observe an overall effect of prenatal by postnatal treatment interaction (*F*_2,60 _= 0.49; *p *> 0.60), post-hoc analysis of mean following behavior using Newman-Keuls criterion for multiple comparisons revealed interesting differences in behavior among the six treatment combinations. Animals with prenatal exposure to ethanol were found to have followed an intoxicated peer more frequently than animals from either the PF (mean difference = -5.17, *p *< 0.05) and FC (mean difference = -8.17, *p *< 0.01) treatment groups. Animals from the PF and FC groups did not differ from each other (mean difference = -3.00, *p *> 0.05). Further, there was no evidence of an effect of prenatal treatment on following a non-intoxicated peer (ET vs. PF: mean difference = -3.41; ET vs. FC: mean difference = -4.91; PF vs. FC: mean difference = -1.50; all *ps *> 0.05). Importantly, on average, ET animals followed an ethanol demonstrator with a significantly higher frequency than a water demonstrator (mean difference = -6.92, *p *< 0.05). Taken together, our results demonstrate that prenatal ethanol exposure enhanced odor-guided social behavior specifically in response to ethanol odor, increasing following behavior only in the presence of an intoxicated peer.

### Experiment 2: Adult behavioral and neurophysiological assessment

#### Reflexive sniffing behavior

**Figure 7:**
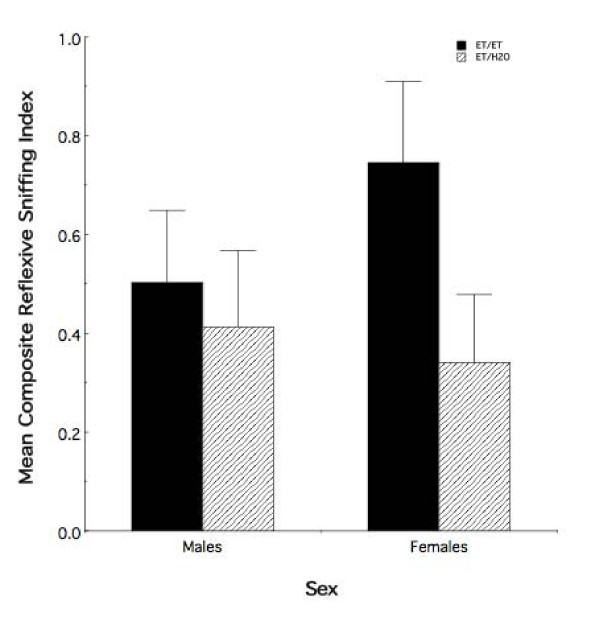
**Fetal and adolescent ethanol exposures, combined, leads to altered behavioral responses in adult females**. Mean composite reflexive sniffing index values (± sem) for all adult observer animals that received prenatal ethanol exposure as a function of sex and adolescent treatment. Relative to prenatal experience alone, subsequent ethanol re-exposure in adolescence altered the response to ethanol odor in adult females. ET/ET = ethanol animals with ethanol exposure in adolescence. ET/H2O = ethanol animals with water exposure in adolescence.

The primary focus was to test the proposition that combined fetal and adolescent exposure perpetuates into adulthood the enhanced behavioral response to ethanol odor observed in infant and adolescent animals. Thus, we evaluated the consequence of adolescent ethanol odor re-exposure, relative to fetal exposure alone, on the behavioral response to ethanol odor in P90 animals (recall that the effects of fetal exposure on olfactory function ameliorate by adulthood [19]). Figure 7 illustrates the mean Composite Reflexive Sniffing Index (± sem) of all adult observer animals having received prenatal ethanol experience as a function of sex and adolescent odor experience (i.e., exposure to an ethanol or water demonstrator). Randomized-blocks ANOVA demonstrated an overall effect of adolescent odor experience in animals that were prenatally exposed to ethanol (F_1,14 _= 9.22, *p *< 0.01) with no overall sex difference (F_1,14 _= 0.10, *p *> 0.70). There was no evidence for an overall sex by adolescent treatment interaction (F_1,14 _= 3.93, *p *= 0.067).

As indicated in Figure 7, however, the overall adolescent treatment effect appears to be driven by an altered behavioral response that is specific to the female ET animals. Post-hoc analysis using Newman-Keuls criterion revealed a significant difference in the behavioral response of female ET animals as a result of adolescent ethanol odor experience relative to ET animals that were water observers (mean difference = 0.40, *p *< 0.05). No such effect was observed in males (mean difference = .09, *p *> 0.05).

**Figure 8:**
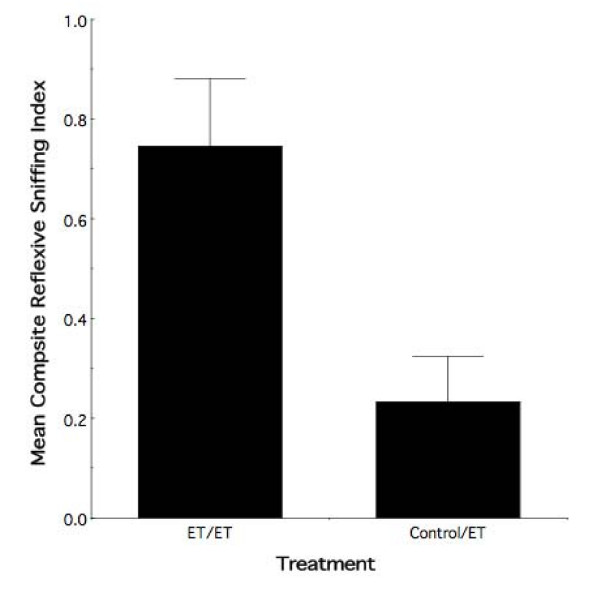
**Combined ethanol exposures leads to behavioral alterations in adulthood as compared with adolescent exposure alone**. Mean composite reflexive sniffing index values (± sem) for all female observer animals that received adolescent ethanol exposure as a function of prenatal treatment. Female animals display a differential effect of prenatal treatment on the response to ethanol odor in adulthood. ET/ET = ethanol animals with ethanol exposure in adolescence. Control/ET = pair-fed and free choice animals with ethanol exposure in adolescence.

To further explore the specificity of the adult female effect, we examined whether the preserved behavioral response to ethanol odor is due to the combination of prenatal and adolescent ethanol exposures or the effect of the most proximal (adolescent) experience. Preliminary evaluation of adolescent control animals (both PF and FC) having received adolescent ethanol odor exposure and those receiving water exposure revealed no evidence of an altered behavioral response to ethanol odor both within and across control conditions when tested as adults (all *ps *> 0.15). In short, adolescent odor exposure alone is insufficient to yield a behavioral effect in adult female animals. Therefore, in the following analysis we combined the PF and FC animals having received ethanol exposure in adolescence into a single control group. As illustrated in Figure 8, exploratory randomized blocks ANOVA of adult observer females (i.e., ET, and controls) that received ethanol exposure in adolescence revealed an overall effect of prenatal treatment (F_1,10 _= 8.46, nominal *p *< 0.02) on the behavioral response to ethanol odor. Taken together, the results of Figures 7 and 8 give strong support the interpretation that combined fetal and adolescent exposure yields persistence of the prenatal effect, alone, into adulthood.

#### Epithelial response

The goal of our optical recording of the OE was to test the hypothesis that re-exposure to ethanol odor during adolescence would lead to the persistence of fetal ethanol induced neurophysiological alterations into adulthood. To test our hypothesis, we evaluated the consequence of adolescent ethanol odor re-exposure relative to fetal exposure alone on the response of the OE to six odorants. As described in the Methods section, the epithelial response to an odorant has both temporal and magnitude components. Thus, the odorant response was characterized using two magnitude response measures and three temporal measures [19]. Using an identical procedure as with our behavioral data, PCA was used to encapsulate the five response measures into a fewer number of uncorrelated measures of the neural response for each odorant. Neither of the resultant factors from the PCA met our predictive error criterion for variable selection (F ≥ 2.0; [19,39]) for either the septum or turbinate OE. As such, this finding indicates that adolescent ethanol re-exposure did not perpetuate the neural effects of fetal exposure observed in infant [19] and adolescent animals [21] into adulthood.

## Discussion

Developmental changes in the brain add to the age-specific behavioral uniqueness of adolescence, including the inherent increased propensity to use drugs [45]. Thus, within the context of the present study a key question is the connection between fetal ethanol exposure, chemosensory plasticity, and adolescence. From a teleological standpoint, it has been argued that animals benefit from chemosensory mechanisms that emphasize the animal's attention to stimuli that are "presumed" significant for survival and reproductive fitness (e.g., the odor/taste of foods eaten by their mother) [6]. This may be particularly true for adolescent animals, which are in the process of separating from their mothers and learning which foods are "safe" to eat. Given that chemical stimuli in a mother's diet can contaminate the fetal environment and provide sensory stimulation [e.g., [7-9]], it has been suggested that it is highly adaptive for developing embryos to tune their chemosensory systems to the molecules present in the amniotic fluid, and feed selectively on specific foods after weaning.

Unfortunately, however, an adaptive mechanism that should work to the advantage of the animal may within the context of fetal ethanol exposure work to its disadvantage. Youngentob and colleagues [19] demonstrated that prenatal ethanol exposure results in an alteration in olfactory system function that is manifested both behaviorally and neurophysiologically in the P15 rat and that these effects, although persistent into adolescence [21], are absent in adults (P90). Notably, fetal exposure results in enhanced ethanol intake in P15 rats [20] that is causally related to both the altered response to ethanol odor as well as altered gustatory perception [Youngentob and Glendinning, submitted]. These effects are also absent in adult littermates. The ages at which these studies observed a consequence of fetal exposure are in keeping with earlier literature on early postnatal [e.g., [14,17,18,46]] and adolescent ethanol intake effects [22-24], and at least one adult study that used a similar gestational exposure model and studied ethanol intake at similar early and late developmental ages [47]. Thus, the available data point to adolescence as a potentially critical transition point for perpetuating the olfactory experience-induced effects of fetal ethanol exposure into adulthood. Such a proposition is clinically relevant since, as previously noted, adolescence is a key transition point for emergent patterns of ethanol abuse and especially so following prior fetal exposure [45].

Social interaction has been shown to play an important role in adolescent decision-making and is highly correlated with ethanol use in adolescence [35]. In the present study, we utilized a well-established social transmission of food odor preference paradigm [30-32] to provide a second ethanol odor experience during adolescence. This paradigm centers on the finding that rodents obtain information regarding foods to ingest based, at least in part, on an interaction with a known peer that has recently ingested a "novel" diet. In other words, olfactory cues perceived on the breath of another animal (i.e., the demonstrator in our study) are known to impact later food choices of a conspecific (i.e., the observer). Importantly, several studies have demonstrated that both naïve infant and adolescent observer animals will increase their ethanol intake as a consequence of interaction with a peer that was administered ethanol [e.g., [33,34,48,49]]. Thus, this procedure permitted us to assess a second chemosensory-related ethanol experience through a potentially relevant form of human adolescent behavior, namely, social interaction with an exposed (i.e., intoxicated) peer [35].

The results of our study significantly extend upon the prior work investigating experience-induced olfactory plasticity in response to fetal ethanol exposure [[19,21], Youngentob and Glendinning, submitted]. Behavioral testing in late adolescence demonstrated that: (1) adolescent re-exposure to ethanol odor augments the known olfactory response resulting from fetal experience with the drug; (2) when tested in adolescence, the consequence of adolescent ethanol odor exposure in control animals results in an enhanced olfactory response to ethanol odor similar to those resulting from fetal exposure alone; and (3) those animals receiving combined fetal and adolescent experience with ethanol odor demonstrate an augmented behavioral response to ethanol odor relative to those animals with either one developmental experience (i.e., fetal or adolescent) or no experience with ethanol. Taken together, the results clearly demonstrate a cumulative consequence of the number of developmental time points during which ethanol experience occurs on the response of adolescent animals to ethanol odor.

Testing in adulthood revealed alterations in the behavioral response to ethanol odor only in females having experienced the combination of fetal and adolescent ethanol exposures. No behavioral effects were found in the adult males as a consequence of previous exposures to ethanol odor. Based on our adolescent results illustrating no differential effect of sex on the behavioral response to ethanol, we did not anticipate sex differences to be present in adulthood. Nonetheless, our findings are not overly surprising given the variable evidence in the literature regarding the differential effects of sex on the responsivity to a substance following exposure in a social situation. While several studies do not report an effect of sex when examining both male and female observers [e.g., [31,49,50]], others strongly suggest sex may play a critical role in the effects of social interaction. Alterations in voluntary ethanol intake due to familiarity with the demonstrator appear to be sex dependant [51], as do changes in food preference following administration of a benzodiazepine anxiolytic to reduce social aggression [52]. Areas of social interaction behavior such as play fighting, which were not analyzed in the context of this experiment, have also illustrated sex differences, with males showing increased play fighting in adolescence as compared to females of the same age [53] While the present study consisted of familiar, same sex littermates, it is not unrealistic to consider that the information received by the observers, through the social transmission of food preference design, could have been altered in a sex dependent manner due to age related changes in social behavior.

Sex dependant alterations in the demonstrators' level of ethanol intoxication could also have contributed to the differential adult effect as well. Although all demonstrators were infused with the same dose of ethanol, recent work in a collaborating lab reveals that adolescent female Long-Evans rats produce a much higher blood alcohol level (BAL) than males after ingesting the same dose of ethanol (F.A. Middleton, personal communication, September 13, 2008). Thus, a higher BAL in the female animals may have resulted in a different level of ethanol odor on the breath of females relative to males. This, in turn, would impact the transmission of the food preference leading to persistence of the behavioral effects into adulthood in the females.

Although the enhanced behavioral response to ethanol odor remains present in adult females, we did not find a persistence of the fetal ethanol induced alterations in the response of the OE for either sex. In considering this finding several issues need to be considered. First, new neurons are continuously integrated into the functional network of the olfactory system (both olfactory sensory [OSN] and olfactory bulb neurons) throughout development and even in adulthood [54-56]. Indeed, expansion of the OE occurs continuously between adolescence and adulthood [56]. Thus, it is possible that as this normal expansion occurs the alteration in the response of the OE to ethanol odor, as a consequence of fetal exposure, became diluted (in short, a potential signal to noise problem) since it is unlikely that the initial fetal exposure effect modified the OE's progenitor cell population [57,58]. Second, our adolescent ethanol exposures were relatively brief (30 minutes), occurring only 4 times, with non-exposure days interposed. As such, based on previous work demonstrating the need for extensive long-term odor exposure to yield an observable effect on the response of the OE [e.g., [42,44]] it is not surprising, in retrospect, that our procedure did not yield additional detectable changes in the OE response.

It is also possible that the alterations in the response of the OE, while causally important in the initial priming of the enhanced behavioral odor effect [19], is not required for behavioral persistence into adulthood. That is, the early tuning of the OE to ethanol odor may reflect a global plasticity response of the olfactory system to ethanol odor that results in effects on more centrally olfactory structures that can be stabilized by adolescent odorant exposure. For example, it has been suggested that neurogenesis in the olfactory bulb plays a key role in information processing and memory storage [54]. With this in mind, we hypothesize that a memory for ethanol has been formed by the alterations in the olfactory system due to prenatal ethanol odor exposure, and that this memory is reinforced by the adolescent ethanol exposure, leading to behavioral manifestations in the adult female.

Finally, with regard to our evaluation of the social interactions, we found that adolescent animals exposed to ethanol prenatally follow an intoxicated peer more frequently than control animals and that this increased behavior was specific to the peers that had ingested ethanol. This subsidiary finding further highlights the experience-induced consequences of fetal ethanol exposure on olfactory-mediated behaviors. Moreover, they suggest that within the context of "at risk" adolescents, prior exposure to ethanol may, among other things, augment the consequences of ethanol related social interaction by increasing the propensity to engage such setting. As such prior fetal exposure may play a key role in the individual affecting its own level of "re-exposure".

In view of the forgoing results, it is important to consider their broader implications to the clinical progression of developing patterns of ethanol abuse. As we have noted, within the context of fetal ethanol exposure, the normal adaptive process of chemosensory plasticity may work to the disadvantage of the adolescent animal: enhancing the already age inherent probability of initial ethanol intake and continued choice behavior in adolescence. Consistent with the implications of our behavioral findings others have shown that the interaction of pre- and postnatal exposure can yield enhanced ethanol avidity relative to the effect of prenatal exposure alone [e.g., [13,59]]. However, if this second experience does not occur, the available data demonstrate that the fetal effects on olfactory function and ethanol avidity will be absent in adults [e.g., [19,20]]. Thus, the timing of re-exposure to ethanol appears critical for persistence of the initial fetal effects into adulthood. In keeping with this hypothesis, the human data show an inverse relationship between the age of first adolescent experience and long-term abuse. Mechanistically, our proposed scenario would result from the alternative maintenance or amelioration of ethanol-induced stimulus activity. Indeed, it is a well-studied observation in olfactory development and even adulthood that stimulus-activated neurons are stabilized and survive while inactive ones are compromised and eliminated [60-62]. Given that this type of highly relevant competition is widespread throughout the developing nervous system [e.g., [63-67]] and adolescence is a dynamic developmental period [45], adolescent re-exposure may be a key element in developing patterns of progressive ethanol abuse.

## Conclusion

The present study adds to the growing body of evidence demonstrating the important relationship between the behavioral responsiveness to ethanol odor and experience-induced modulations in olfactory function in response to the drug. Further, this study highlights a key association between fetal and adolescent experience that appears critical to the progressive nature of developing patterns of continued ethanol abuse.

## Competing interests

The authors declare that they have no competing interests.

## Authors' contributions

All authors were involved in the design of the study; AME and LMY executed the study; AME, PRS, and SLY participated in analysis and interpretation of the data; and AME and SLY prepared and edited the manuscript.
